# Anthropogenic influence on extremes and risk hotspots

**DOI:** 10.1038/s41598-022-27220-9

**Published:** 2023-01-02

**Authors:** Francisco Estrada, Pierre Perron, Yohei Yamamoto

**Affiliations:** 1 Instituto de Ciencias de la Atmósfera y Cambio Climático, Universidad Nacional Autónoma de México, Ciudad Universitaria, Circuito Exterior, 04510 Mexico, DF Mexico; 2 Institute for Environmental Studies, Vrije Universiteit, Amsterdam, The Netherlands; 3 Programa de Investigación en Cambio Climático, Universidad Nacional Autónoma de México, Ciudad Universitaria, Circuito Exterior, 04510 Mexico, DF Mexico; 4 Department of Economics, Boston University, 270 Bay State Rd., Boston, MA 02215 USA; 5 Department of Economics, Hitotsubashi University, 2-1 Naka, Kunitachi, Tokyo, 186-8601 Japan; 6 Tokyo Institute of Technology, Tokyo Tech Academy of Energy and Informatics, Tokyo, Japan

**Keywords:** Climate sciences, Climate change, Attribution, Climate-change impacts

## Abstract

Study of the frequency and magnitude of climate extremes as the world warms is of utmost importance, especially separating the influence of natural and anthropogenic forcing factors. Record-breaking temperature and precipitation events have been studied using event-attribution techniques. Here, we provide spatial and temporal observation-based analyses of the role of natural and anthropogenic factors, using state-of-the-art time series methods. We show that the risk from extreme temperature and rainfall events has severely increased for most regions worldwide. In some areas the probabilities of occurrence of extreme temperatures and precipitation have increased at least fivefold and twofold, respectively. Anthropogenic forcing has been the main driver of such increases and its effects amplify those of natural forcing. We also identify risk hotspots defined as regions for which increased risk of extreme events and high exposure in terms of either high Gross Domestic Product (GDP) or large population are both present. For the year 2018, increased anthropogenic forcings are mostly responsible for increased risk to extreme temperature/precipitation affecting 94%/72% of global population and 97%/76% of global GDP relative to the baseline period 1961–1990.

## Introduction

Understanding how extreme events are changing in a warming world is important from a scientific perspective and for their societal and political impacts^1^. High-impact extreme weather events can change risk perceptions faced by decision-makers and the public view about climate change. Hence, it can influence climate policy and foster more ambitious mitigation and adaptation goals^2–4^.

Changes in precipitation and temperature patterns and the frequency and magnitudes of extreme events are influenced by several thermodynamic and dynamic processes affected by climate change^5,6^. For instance, as warming increases, the capacity of the atmosphere to hold moisture increases and, accordingly, extreme rainfall can exceed past record levels. Estimated trends in daily extreme precipitation events agree with the theory stating that a 7% increase in atmospheric moisture is predicted per degree Celsius increase in global temperature^7,8^. Note that this relationship is not constant and can vary depending on the timescale^9^.From a dynamic perspective, differential warming across regions and feedback processes can have worldwide effects on regional weather and climate, including temperature and precipitation extremes^5,10,11^. For instance, warming in the Arctic is between twice and fourfold as the global average due to local and remote feedback processes broadly labelled as the Arctic Amplification (AA)^12^, while over the last three decades parts of the midlatitudes showed no-warming or even cooling^13,14^. This Warm Arctic Cold Continents/Eurasia (WACCE) pattern decreases the thermal contrast between the Arctic and mid-latitudes affecting the weather and climate of the northern hemisphere’s mid-latitudes through changes in storm tracks, the jet stream and planetary waves^13^. These differences were associated with increases in the occurrence of severe winters, extreme heat in summers and record-breaking precipitation events in the northern hemisphere since the 1990s^15^. Several studies proposed that changes in the atmospheric circulation, in particular a weakening of the northern hemisphere’s jet stream, can produce stagnant weather patterns and more persistent weather extremes^13,16^. This induces higher probabilities of atmospheric blocking and the occurrence of persistent heat waves and extreme rainfall events^16^. A recent study linked WACCE to the spatial pattern of anthropogenic warming on temperature changes, providing an explanation for WACCE and the observed changes in extreme events in mid-latitudes^17^. Differential warming between hemispheres also has important implications on precipitation patterns in the tropics and changes in the frequency and magnitude of extreme events by changing key features of the intertropical convergence zone (ITCZ); e.g., location, width, and circulation strength^18^. Changes in tropical cyclone activity and characteristics also have large impacts on total local rainfall and extreme precipitation events^19^. An event-attribution study of the 2020 North Atlantic hurricane season suggests that anthropogenic warming could account for 5%-10% increase in tropical storms and 8–11% in rainfall rates from hurricane storms and extreme 3-day accumulated rainfall amounts^20^.

The properties of extreme events make their study and characterization particularly challenging. First, by definition, they are rare and the processes are, generally, nonstationary^21–23^. Second, data limitations are present such as small samples, incomplete spatial coverage, as well as inhomogeneities, making difficult detecting, characterizing and attributing trends and other features^24–27^. To address such problems, methods are being adapted and developed to analyze extreme events time series, including extreme value theory modelling^8,28,29^, trend testing and estimation^28,30–32^, and methods to detect structural breaks^33^. Approaches related to optimal fingerprinting techniques, combining physical models’ output and observations, were used to infer the contribution of anthropogenic factors to changes in extreme values^6,34,35^. Concerning data availability, increases in the spatial coverage and time span of observational records were obtained; e.g., the constant updating of international gridded datasets that merge quality checked records from weather stations across the world^36–38^, and the growing availability of reanalysis datasets^39,40^.

The contribution of the Working Group I to the IPCC’s Sixth Assessment Report concludes that (a) the rise in weather and climate extremes already led to irreversible impacts on natural and human systems, (b) it is virtually certain that hot extremes have become more frequent and intense; (c) the same applies to heavy precipitation over most land area since the mid-twentieth century^41^. For temperature extremes, several studies showed that significant warming trends are widespread and attributable to anthropogenic warming, with conclusions robust to different datasets and methods^32,42–45^. Because of differences in local forcing factors like changes in land use (e.g., cropland and irrigation intensification) and aerosols, as well as in circulation patterns, temperature extremes vary across regions. These factors can alter the anthropogenic warming signal at local to regional scales^46–48^.

Changes in the total annual 1-day and 5-day maximum precipitation (Rx1day; Rx5day) are similar over the global, continental and regional scales with statistically significant increases over global land, North America, Europe and Asia^28,31,41^. Changes in heavy precipitation are likely dominated by anthropogenic forcing, although the sign and magnitude depend on location and timescales^49^. Accordingly, their spatial patterns are more heterogeneous compared to temperature changes^5,6,41^. This is partly due to a larger influence of local forcing factors like aerosols and land use change over extreme precipitation, notably in urban areas^50,51^. However, greenhouse gases are considered the dominant contributor to the intensification of extreme precipitation^41,49^. At the regional and local scales, attribution results are less robust because variability and local forcing factors induce a weak signal-to-noise ratio^41,52^.

Studies found evidence about greenhouse gases and aerosol forcing effects on extreme precipitation over North America, Eurasia, in the mid/high latitudes and the global dry regions, and that these effects are different for the two types of forcing^34,53,54^. Globally, minimum temperatures are warming faster than maximum ones; the coldest night increased about 4 °C since the mid-twentieth century, while the hottest day increased 1 °C, compared to the 1960s-1970s period^31,36^.

The attribution of changes in extreme temperature and precipitation mostly depends on climate models’ projections, making the results dependent on the models’ adequacy to reproduce climate and its extremes. The regional performance of global climate models significantly improved, but limitations persist at the regional level^55,56^. Moreover, there are differences between what extreme events represent at the grid-cell level in climate models compared to actual observations^36^. A recent study established the effects of anthropogenic climate change on extreme precipitation using global mean temperatures in an observation-based analysis^28^, suggesting attribution results are robust.

However, global temperatures are a composite response to changes in natural and anthropogenic forcing and the influence of natural variability oscillations, making difficult separating the effects of anthropogenic forcing^57–59^. We adopt an approach using total radiative forcing as covariate in a non-stationary generalized extreme value (GEV). The main contributions of this paper are that it allows for separation of the influence of natural and anthropogenic factors on extreme temperatures and precipitation, and that it documents how the changes affect the risk faced by population and Gross Domestic Product (GDP) metrics. We report maps showing how the risk from temperature and precipitation extremes faced by global population and GDP have changed. We separate these changes in their natural and anthropogenic components.

## Results

### Brief description of data and methods

The data used are yearly series obtained from the HadEX3 dataset: the temperature of the hottest day (TXx), the maximum precipitation in a given day (Rx1day) and over 5 days (Rx5day). The data are for global land in a grid with a spatial resolution of 1.875° longitude and 1.25° latitude, for the period 1901–2018. See methods for details on the treatment of missing data and interpolations. The results for Rx1day and Rx5day are similar; hence, we report those for Rx1day only.

The main tool used is a GEV model with time-invariant shape and scale parameters and time-varying location parameters. All parameters can change across grids. Preliminary statistical investigation showed a good approximation. The variation of the location parameter is a function of the main components of total radiative forcings: greenhouse-gases, anthropogenic aerosols and natural (solar and stratospheric aerosols). This allows for separation of the effect of each component on the measures investigated, namely: a) the probabilities that the values in 2018 exceeds the average of the reference period 1961–1990; b) the return level defined as the probability that the value in 2018 exceeds some threshold be 0.1, from which we can infer the highest value of TXx or Rx1day that occurs once in ten years, with some probability. We compute counterfactuals attributing changes in probabilities according to anthropogenic and natural factors. See methods for details on the estimation and the definition of the forcing variables used.

### Observed changes in the probabilities of occurrence and return levels of extreme events

**Figure 1:**
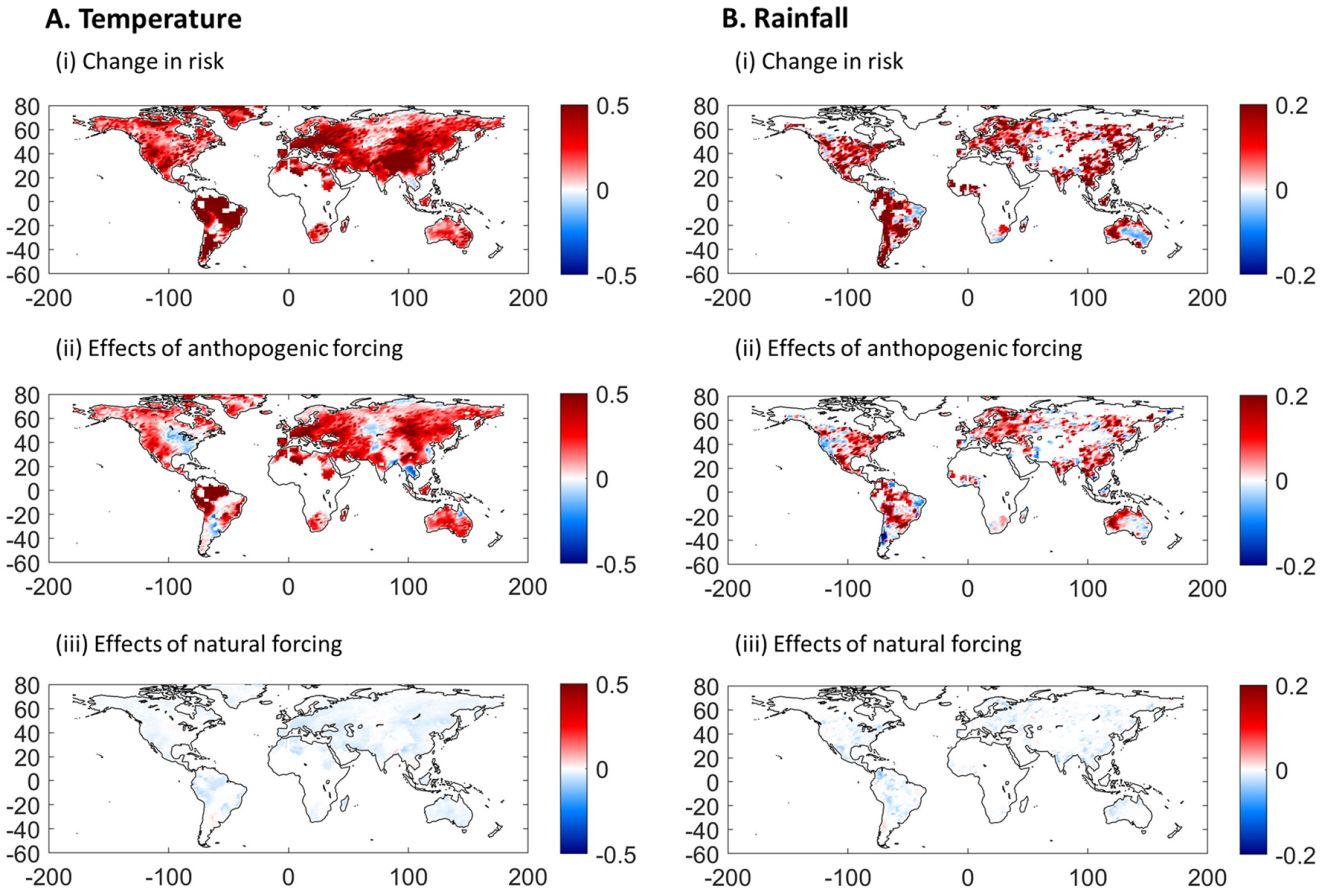
Changes in the risks of extreme temperature and rainfall and the contributions of natural and anthropogenic radiative forcing. Panels (**A** and **B**) show the results for extreme temperature and rainfall, respectively. Subpanels (i) show the change in the probabilities of exceeding the 90th percentile of the annual maximum values calculated for the reference period 1961–1990 and evaluated for 2018. Subpanels (ii) and (iii) show the contributions of such changes induced by anthropogenic and natural forcing, respectively. This figure was created using MATLAB R2020a (https://www.mathworks.com/).

The first set of results are in Fig. 1; Panel A for temperatures (TXx) and Panel B for precipitation (Rx1day). Subpanels (i) show the probabilities in 2018 of exceeding the 90th percentile of the annual maximum values, $\mathrm{Pr}({x}_{i,2018}\ge {q}_{.9, 1961-1990})$ minus 0.1 for the reference period 1961–1990. If TXx and Rx1day were stationary series, $\mathrm{Pr}\left({x}_{i,2018}\ge {q}_{.9, 1961-1990}\right)-0.1=0$. However, the results show large increases in the risk of extreme temperature for most of the continent land with available observations. Areas in dark red depict where these probabilities have increased at least five-fold by 2018, relative to the base-period 1961–1990. These include most of South and Central America, southern and northern Mexico, central and west North America, Europe, Middle East, and East Asia. A few regions in North America (eastern Canada and northeast US), South America (Bolivia, Paraguay, and Uruguay), Russia and Southeast Asia have exceedance probabilities increasing by less than 50% or decreasing.

Anthropogenic forcing alone has increased the risk of extreme temperature in most land regions worldwide. For most of Europe, the Middle East, North and South Africa, Asia and Mexico, central and western US, most of South America and Australia, the probabilities of extreme temperature at least doubled with an increase of about 2 W/m^2^ by 2018 in anthropogenic forcing (subpanel (ii) of Fig. 1A). Over large areas of Russia and Canada, as well as in Norway, Sweden, Argentina and parts of Australia and China, the probabilities of exceeding the 90^th^ percentile in TXx over the reference period increased (in some cases nearly doubled) with anthropogenic forcing. Some exceptions with decreased probabilities induced by anthropogenic forcing include the central/southeastern part of the US, particularly the “warming hole”, associated with the local effects of aerosols over the region^60,61^; others are parts of Eurasia, Southeast Asia and Argentina. Note, however, that the model’s coefficients associated with radiative forcing in such regions are typically not statistically significant. In most regions, the contribution of natural forcing to changes in the risk of extreme temperature is weak, if any (subpanel (iii) Fig. 1A). Anthropogenic forcing is clearly the dominant component of total radiative forcing and the results using the latter are similar and not reported. Figure S1 shows the yearly change in the probabilities of extreme temperature for the period 1901–2018. Since the late 70 s, uninterrupted and widespread increases in the risk of extreme temperature in most land regions have occurred. This period is consistent with the dates previously identified for the climate system’s response to increases in anthropogenic forcing (particularly when natural variability is not filtered out)^57,62^.

We now discuss results concerning extreme precipitation in Fig. 1 (Panel B). Increases in the risks of extreme precipitation are also widespread across all global land areas although the spatial patterns are more heterogeneous. In all continents with sufficient observations, the probabilities of Rx1day exceeding the 90th percentile of the reference period increased at least 50% by 2018, with many areas showing three-fold increases. Large decreases in the risk of extreme precipitation occurred in the central-southeast of Australia, where the probability of exceedance is close to zero. The contribution of anthropogenic forcing to increasing the risks of extreme precipitation is particularly large (about three-fold) in northwestern Australia, some regions of South America (Colombia, Ecuador, northwestern and southeastern Brazil, Bolivia, Paraguay and Uruguay), central and northwest of Mexico, Eastern Europe, and some parts of South Asia (subpanel (ii) of Fig. 1B). Increases in the probabilities of exceedance near 50% associated with the rise in anthropogenic forcing occurred for much of North America, Europe, Asia and Australia, with much larger increases in some parts. In contrast, important reductions occurred along the Pacific coast of South America. As in the case of extreme temperature, the contribution of natural forcing to changes in the risk of extreme precipitation is small and mostly negative and the probabilities of extreme precipitation increased since the late 70 s in a widespread manner (Figure S2).

An important feature of these results is that due to nonlinear effects, the total changes in risk in both extreme temperature and precipitation is much larger than those attributable to anthropogenic and natural forcing alone. Hence, large changes in risk can occur with even small increases in anthropogenic forcing, which can amplify the effects of variations in natural factors. With a higher level of anthropogenic forcing, variations in natural factors can trigger even more extreme events.

**Figure 2:**
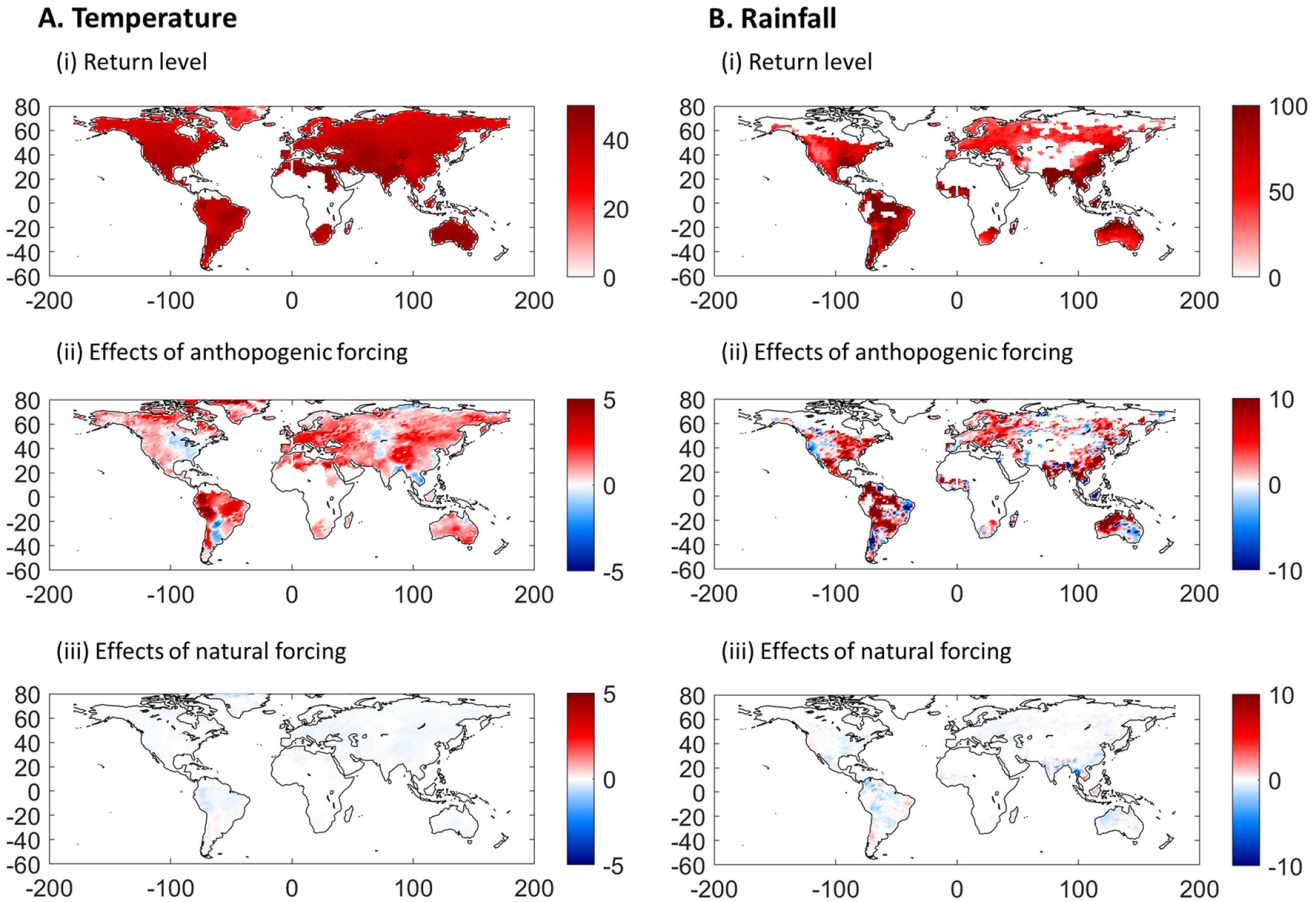
Return levels of extreme temperature and rainfall and the contributions to natural and anthropogenic radiative forcing. Panels (**A** and **B**) show results for extreme temperature and rainfall, respectively. Subpanels (i) show the return levels for the annual maximum that occur once in ten years. Subpanels (ii) and (iii) show the contributions to these return levels in 2018 of the anthropogenic and natural forcing, respectively. This figure was created using MATLAB R2020a (https://www.mathworks.com/).

Subpanels (i) of Fig. 2 shows the estimated return levels of extreme temperature and rainfall, defined as the highest value of TXx or Rx1day occurring once in ten years (see Methods). The highest return levels in TXx are mostly in tropical and subtropical regions of the northern hemisphere and in Australia, while in the case of Rx1day they occur in South America, tropical cyclone, and monsoon regions. The contribution of anthropogenic forcing (Subpanels (ii)) to these return levels is spatially heterogeneous, particularly in the case of rainfall. The largest increases in TXx associated with anthropogenic forcing (3–5 °C) are near the Amazon rainforest, Bolivia, Peru, and Brazil, as well as near the Arctic and in the high latitudes of North America. For Central and Eastern Europe, Northern Africa and some parts of Eastern Asia the evidence also shows important contributions of anthropogenic forcing to the return values of TXx (about 2.5 °C). For Rx1day, these occur in the northeastern part of Australia, Southeast Asia, South America, central and northern Mexico, the east of the US and Canada, and in Eastern Europe. The contribution of natural forcing to changes in the return levels of both types of extremes is near zero (Subpanels (iii)).

### Hotspots of changes in risk of extreme temperature and precipitation and high levels of exposure

Of interest when studying how climate change alters extreme events are the societal impacts^41,63,64^. Changes in extreme events affect disaster risk, but the interaction with exposure and vulnerability patterns drives disasters and socioeconomic losses^1^. To better understand the potential consequences of the reported changes in the probabilities of extreme values of TXx and Rx1day, we use global population and GDP maps to represent exposure. We evaluate the locations of the socioeconomic indicators subject to increasing levels of risk associated to anthropogenic climate change (Table 1; see Methods). The population and GDP data used are for the year 2018.

**Table 1:** World population and GDP with increasing risk of extreme temperature and rainfall.

Population (million)			GDP (billion US dollar)		
*A. Temperature*					
World total	7380	–	World total	92,835	
Data available	5912	(100.0%)	Data available	82,453	(100.0%)
*Area of increasing risk from 1961–90:*			*Area of increasing risk from 1961–90:*		
	5584	(94.4%)		79,912	(96.9%)
*Area of increasing risk by forcing:*			*Area of increasing risk by forcing:*		
Total forcing	4281	(72.4%)	Total forcing	63,337	(76.8%)
Natural forcing	1547	(26.2%)	Natural forcing	18,562	(22.5%)
Anthropogenic forcing	4281	(72.4%)	Anthropogenic forcing	63,337	(76.8%)

Population (million)			GDP (billion US dollar)		
*B. Rainfall*					
World total	7380	–	World total	92,835	–
Data available	5478	(100.0%)	Data available	76,895	(100.0%)
*Area of increasing risk from 1961–90:*			*Area of increasing risk from 1961–90:*		
	3958	(72.2%)		58,442	(76.0%)
*Area of increasing risk by forcing:*			*Area of increasing risk by forcing:*		
Total forcing	3719	(67.9%)	Total forcing	55,030	(71.6%)
Natural forcing	1386	(25.3%)	Natural forcing	17,273	(22.5%)
Anthropogenic forcing	3715	(67.8%)	Anthropogenic forcing	55,003	(71.5%)

Sections A and B show the estimates of world population and GDP facing increasing risk from extreme temperature and rainfall, respectively. The first part in each panel shows the amount of population and GDP in grid cells for which there was sufficient data to perform the analysis. The second part summarizes the absolute numbers and the proportions of population and GDP exposed to higher risk from extreme temperature and rainfall. The third part presents estimates of the absolute numbers and the proportions of population and GDP with higher risk associated to natural and anthropogenic forcings.

In the case of extreme temperature (TXx), about 80% of the global population in 2018 (7380 million people) were located in grid cells with sufficient information to fit the GEV model. About 5,912 million people, 94.4% in 2018 faced an increased risk from extreme temperature in comparison with the reference period (1961–1990), and about 72% in areas with increasing risk associated with rise in anthropogenic forcing. In contrast, only 26% of the population faced increased risk from temperature related to changes in natural forcing. About 89% of global GDP in 2018 was located in grid cells where the GEV model could be estimated. From this group, approximately 97% were exposed to increasing risk from temperature, with about 77% and 23% of global GDP facing higher risk associated with changes in anthropogenic and natural forcings, respectively. The majority of global population and the highest share of GDP experienced increasing risks from extreme rainfall. About 74% and 83% of global population and GDP in 2018 were located in grid cells that had sufficient Rx1day data for the analysis. About 72% and 76% of global population and GDP, respectively, faced higher risk from extreme precipitation in 2018 compared to the 1961–1990 reference period. The proportions of population and GDP with higher risk from extreme rainfall associated with anthropogenic forcing are 68% and 72%, respectively, with only 25% and 23% related to natural forcing.

**Figure 3:**
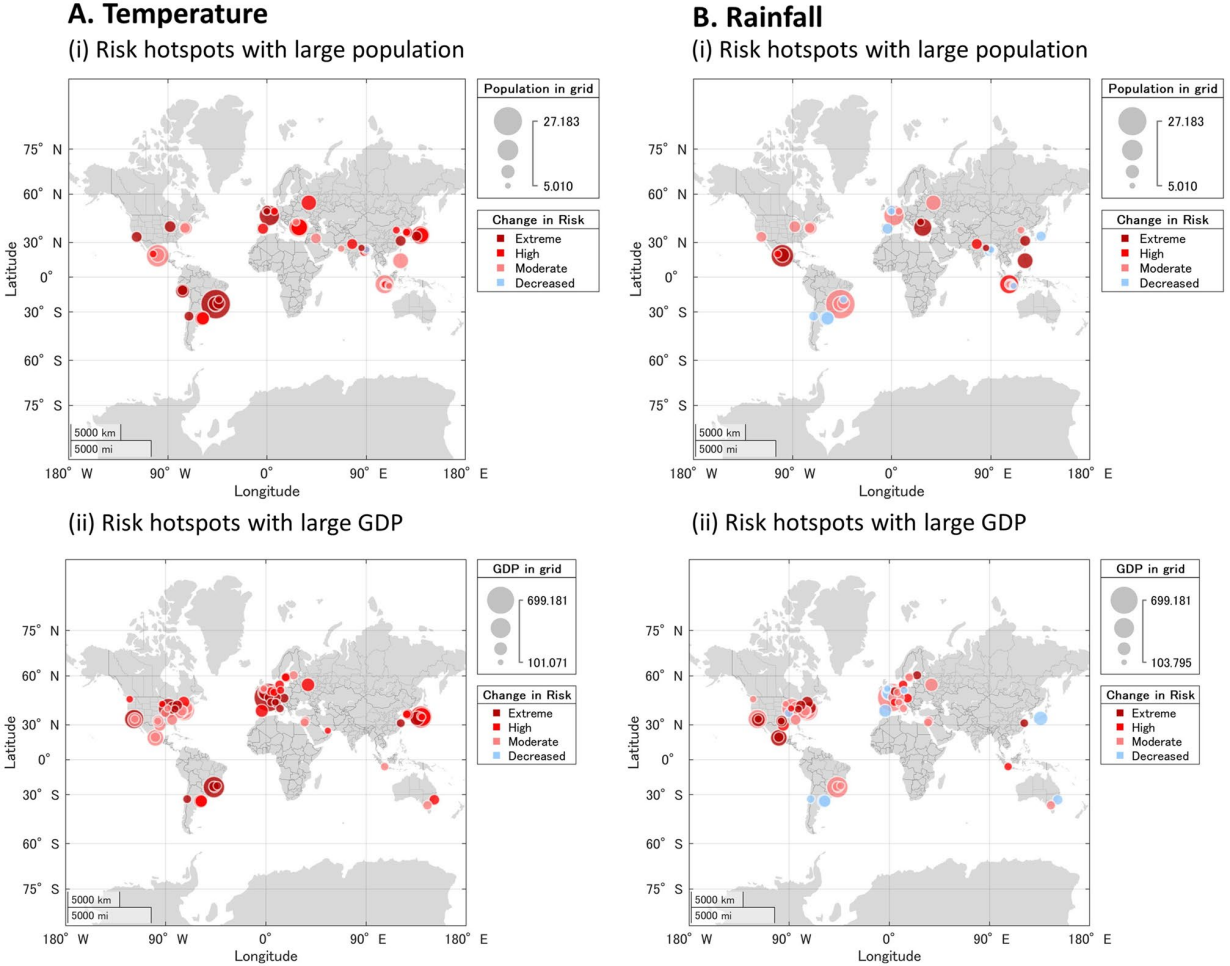
Risk hotspots of increased probabilities of exceedance in extreme temperature and rainfall and high population and GDP exposure. Bubbles are shown for grid cells for which large changes in risk and high levels of population and GDP are present. The size of the bubble represents the amount of population or GDP exposed in the grid, while the color depicts how risk has changed (probability in 2018 minus 0.1) with respect to the 1961–1990 reference period. Panel (**A**) shows the results for extreme temperature, where light blue represents decreases in risk, while pink, red, and dark red indicate moderate (0–20%), high (20–40%) and extreme (> 40%) increases in risk, respectively. Panel (**B**) shows the results for extreme rainfall, where light blue represents decreases in risk, while pink, red, and dark red indicate moderate (0–10%), high (10–20%) and extreme (> 20%) increases in risk, respectively. This figure was created using MATLAB R2020a (https://www.mathworks.com/).

Figure 3 shows risk hotspots, defined as regions with large changes in the probabilities of both extremes and risk exposure. They consist mostly of large cities worldwide. They account for more than 50% of the global population and 80% of global GDP^65,66^. In the case of extreme temperature/population, hotspots are large cities in South America (Rio de Janeiro, Lima, Santiago), the US (California and Chicago), Europe (Paris, London), China (Shanghai), Japan and Indonesia. Hotspots of extreme temperature/GDP are more common and include most large cities in Europe, the US northeast and California, Japan, and Brazil. There are almost no hotspots with decreasing probabilities of extreme temperature. Extreme hotspots of rainfall population/GDP are mainly located in Mexico (Mexico City, Guadalajara), the US (California, Houston, and cities in the northeast), Europe (Amsterdam, Helsinki, Bucharest), and China (Shanghai). There are some locations with high levels of exposure for which the risk of extreme precipitation has decreased, such as in South America (Buenos Aires, Santiago), Europe (Madrid, London), Japan (Osaka), and Australia (Sydney).

## Discussion

Anthropogenic influence with the climate system has already altered central characteristics of extreme events, including their frequency and intensity. Our results show that the risks of extreme temperature and precipitation events has increased in most of land up to five- and three-fold in the case of TXx and Rx1day, respectively. Moreover, such increases in risks have risen since the late 70 s and very rapidly over the past three decades. Nonlinear effects characterize the total changes in risk in both extreme temperature and precipitation and can produce large changes in risk even for small increases in anthropogenic forcing or changes in natural factors. This is particularly worrisome since, as our results show, high population and GDP exposure areas tend to correspond with increases in risk from temperature and precipitation. Such areas are mainly urban where local factors such as the Urban Heat Island effect (UHI) are likely contributing to the increasing trends in extremes. The synergy between global and local (due to UHI) climate change in cities can exacerbate socioeconomic losses and contribute to more severe heat waves and precipitation events^50,51,67,68^. Given the socioeconomic importance of these findings, it would be valuable to extend the proposed methodology to study the attribution of extreme events at the city scale and to evaluate the relative contribution of UHI effects^69^. The results presented here provide further support for adopting strategies to reduce the UHI effect, as they can be low-cost risk reduction instruments for large shares of global population and GDP with increasing risks from climate change.

## Methods

### Data

The extreme temperature and precipitation indices TXx, Rx1day and Rx5day were obtained from the HadEX3 dataset^37^ (available at https://www.metoffice.gov.uk/hadobs/hadex3/). This dataset contains 29 indices of temperature and precipitation^70^ for global land in a grid with a spatial resolution of 1.875° longitude and 1.25° latitude, for the period 1901–2018. The indices were interpolated to a 0.5ºx0.5º grid that matches the population and GDP maps obtained from the CLIMRISK integrated assessment model^71–73^. These socioeconomic scenarios are consistent with the SSP5 scenario^74^ and the estimates used in this paper represent population and GDP (US$2005) in year 2018. Radiative forcing time series are from the Goddard Institute for Spatial Studies (GISS) and were produced for NASA’s CMIP6 simulations^75^ (https://data.giss.nasa.gov/modelforce/). All radiative forcing variables are expressed in W/m^2^ and encompass the historical period (1850–2014) extended to 2018 using the SSP2-4.5 scenario. The dataset contains estimates of the well-mixed greenhouse gases (WMGHG; CO_2_, CH_4_, N_2_O, CFC-12, and CFC-11, plus a radiative equivalent of 38 other long-lived GHGs); natural forcing (NAT; solar, volcanic aerosols, orbital); the total radiative forcing (WMGHG, NAT, O3, the direct and indirect effects of aerosols, land use and irrigation). We define anthropogenic forcing as the sum of WMGHG and the direct and indirect effects of tropospheric aerosols.

### Statistical methods for detection and attribution of climate change

The use of observation-based and time series methods in detection and attribution studies is frequent in the literature^57,76–81^. These methods do not depend on the accuracy and performance of complex climate models, but attribution does require invoking a physical model that may be implicit in the statistical framework used^82^. The present analysis is grounded on a zero-dimensional energy balance model^57,83^ and on the generalized extreme value (GEV) model to estimate the probabilities of extreme events^21,84^.

Previous work provided the basis for the use of time series models to analyze the relationships between radiative forcing and temperature^83,85^. A basic representation of these time series models is:

1 $${T}_{t}=\alpha +\gamma {F}_{t}+{\varepsilon }_{t},$$

where ${T}_{t}$ is global temperature, ${F}_{t}$ is a measure of the change in radiative forcing, $\alpha$ and $\gamma$ are the intercept and slope parameters, respectively, and ${\varepsilon }_{t}$ is a stochastic noise process that represents natural variability^57^. A simple two-compartment climate model^57,83,86,87^ can provide a representation of the structural model supporting Eq. (1). This climate model has an upper compartment (*U*) and a lower compartment (*L*). The first mainly represents the atmosphere and the upper ocean, and the latter the deep ocean. These components are thermally coupled as follows^83^:

2 $${C}_{U}\frac{d{T}_{U}}{dt}=F-\lambda\Delta {T}_{U}-\beta \left({\Delta T}_{U}-\Delta {T}_{L}\right)$$

3 $${C}_{L}\frac{d{T}_{L}}{dt}=\beta \left(\Delta {T}_{U}-{\Delta T}_{L}\right)$$

where ${C}_{U}$ and ${C}_{L}$ represent the heat capacity of the upper and lower compartments, respectively, while ${\Delta T}_{U}$ and ${\Delta T}_{L}$ are the changes in temperature in the respective compartments. *F* is the external forcing, and $\lambda$ and $\beta$ are the climate response and heat exchange coefficients. The heat capacities differ greatly between compartments, being much larger in the lower than in the upper compartment. This is also the case for the time constant of their responses to changes in radiative forcing. In the case of the upper compartment the time constant is about 4 to 9 years, while for the lower compartment it ranges from 400 to 580 years^83,86^. A large fraction of time series and observational methods for attribution focus on the transient climate response (TCR) which characterizes the response of the upper compartment to sustained changes in the external radiative forcing. The TCR is defined by ${S}_{tr}={\left(\kappa +\lambda \right)}^{-1}$ were $\kappa$ is the heat uptake coefficient of the climate system. This coefficient relates the time-dependent changes in surface temperature and external forcing via $\Delta T\left(t\right)= {S}_{tr}F(t)$ and it is represented by $\gamma$ in Eq. (1)^57^. The response of surface temperatures to external radiative forcing is dominated in the observed period by the short time constant of the upper compartment and the TCR^81,83,87^. This provides a physical explanation for why temperatures and external forcing share a common nonlinear trend and common features such as co-breaks.

Changes in global temperature have been linked to changes in extreme temperature and precipitation events by using global temperatures as a covariate in the location parameter of a GEV model as follows^28^.

4 $${\mu }_{i,t}={a}_{0,i}+{b}_{1,i}{T}_{t}$$

where ${T}_{t}$ is global mean temperature, and ${\mu }_{i,t}$ is the location parameter (see next subsection). As was proposed in previous studies using co-trending methods^57,62,79,81^, ${F}_{t}$ in Eq. (1) provides a representation of the warming trend that is free from the high- and low-frequency natural variability. Imposing ${\varepsilon }_{t}=0$ into Eq. (1) and substituting in (4), we have:

5 $${\mu }_{i,t}={a}_{0,i}+{b}_{1,i}\left(\alpha +\gamma {F}_{t}\right)={\mu }_{0,i}+{\mu }_{1,i}{F}_{t}$$

which is used in this paper to investigate the contributions of different components of ${F}_{t}$ to changes in the probabilities of extreme events.

### Description of estimation of the generalized extreme value model

We use the GEV model to estimate probabilities of extreme events for each geographical grid $i$ in year $t$. Under certain conditions, the sample maximum of continuous random variables has only three possible families of asymptotic distributions, Gumbel, Fréchet, and Weibull^84^. The standard stationary GEV distribution unifies these three families and provides a distribution function of the sample maximum $x$ given by

6 $$G\left(x\right)=\left\{\begin{array}{c}\mathrm{exp}\left\{-{\left[1+\xi \left(\frac{x-\mu }{\sigma }\right)\right]}^{-1/\xi }\right\} \mathrm{if} \xi \ne 0\\ \mathrm{exp}\left\{-\mathrm{exp}\left[-\left(\frac{x-\mu }{\sigma }\right)\right]\right\} \mathrm{if} \xi =0\end{array}\right.$$

defined on the set $\{x:1+\frac{\xi }{\sigma }(x-\mu )>0\}$ ^88,89^. The GEV distribution is characterized by three parameters: the location $\mu$ and the scale $\sigma$ normalize $x$, the shape parameter $\xi$ specifies the tail behavior. In this study, we assume that the scale and shape parameters, ${\sigma }_{i}$ and ${\xi }_{i},$ are specific to the geographical grid $i$ but are time-invariant. The location parameter ${\mu }_{i,t}$ is also specific to the geographical grid but may change over time in relation to the global level of total radiative forcing (${{F}_{t}}_{t}$) so that

7 $${\mu }_{i,t}={\mu }_{0,i}+{\mu }_{1,i}{F}_{t}$$

where the total forcing is the sum of the natural (${NAT}_{t}$) and anthropogenic forcings, the latter being the sum of the greenhouse gases (${GHG}_{t}$) and the effects of aerosols (${AER}_{t}$). Hence,

8 $${F}_{t}={NAT}_{t}+{GHG}_{t}+{AER}_{t }$$

This is a typical formulation in the literature to account for the nonstationarity of the extreme values^21^. See Supplementary Information for a more detailed description of the models and approach used.

## Supplementary Information

Supplementary Information 1.

Supplementary Information 2.

## Data Availability

Data used are publicly available from the original sources https://data.giss.nasa.gov/modelforce/ and https://www.metoffice.gov.uk/hadobs/hadex3/.
