# Supplementary material for: Anthropogenic influence on extremes and risk hotspots
Source: Sci Rep. 2023 Jan 2;13:35. doi: 10.1038/s41598-022-27220-9 (PMC9807642; doi:10.1038/s41598-022-27220-9)
Supplement: Supplementary file 1 — Supplementary Information 1. [file 41598_2022_27220_MOESM1_ESM.docx]

**Anthropogenic influence on extremes and risk hotspots**

Francisco Estrada^1,2,3*^, Pierre Perron^4^ & Yohei Yamamoto^5,6^

^1^Instituto de Ciencias de la Atmósfera y Cambio Climático, Universidad Nacional Autónoma de México, Ciudad Universitaria, Circuito Exterior, 04510 Mexico, DF, Mexico, ^2^Institute for Environmental Studies, Vrije Universiteit, Amsterdam, Netherlands, ^3^Programa de Investigación en Cambio Climático, Universidad Nacional Autónoma de México, Ciudad Universitaria, Circuito Exterior, 04510 Mexico, DF, Mexico, ^4^Department of Economics, Boston University, 270 Bay State Rd., Boston, MA, 02215, USA. ^5^Department of Economics, Hitotsubashi University, 2-1 Naka, Kunitachi, Tokyo, Japan, 186-8601, ^6^Tokyo Tech Academy of Energy and Informatics, Tokyo Institute of Technology, Tokyo, Japan.

*Corresponding author, feporrua@atmosfera.unam.mx

**Supplementary Information**

**Methods**

To estimate the GEV model, we take block maxima with one calendar year as one block and fit the temperature and precipitation data using this model for each geographical grid. We use the TXx and Rx1day data from the HadEX3 dataset to investigate extreme temperature and extreme rainfall. We estimate the model by the method of maximum likelihood using data from January 1901 to December 2018, so that 118 data points from 1901 to 2018 are available for complete series. However, the dataset is subject to missing periods for some grids. To account for this limitation, we consider only the annual maxima for calendar years having observations for at least 9 months. We then estimate the GEV model for each geographical grid having annual maxima for more than 30% of the entire yearly sample 1901-2018.

To estimate the models, we need initial values to initiate the numerical optimization of the log-likelihood function. We use the least squares estimate of $x_{i,t}=\mu_{0,i}+\mu_{1,i}{TOT}_{t}+error$ for $t=1901,\ldots,2018 ,$ where $x_{i,t}$ is the annual maxima of TXx or Rx1day as the dependent variable and ${TOT}_{t}$ is the dependent variable. We use the coefficient estimates as the initial values for $\mu_{0,i}$ and $\mu_{1,i}$ and the sample standard deviation of the least square residuals as the initial value for $\sigma_{i}$. We set the initial value for $\xi_{i}$ to be 0.1 for all $i$. Our results are not sensitive to these choices. Once we obtain the parameter estimates $\hat{\mu}_{0,i}$, $\hat{\mu}_{1,i}$, $\hat{\sigma}_{i}$ and $\hat{\xi}_{i}$, the location parameter is recovered by plugging these values in (2), that is, $\hat{\mu}_{i,t}=\hat{\mu}_{0,i}+\hat{\mu}_{1,i}{TOT}_{t}$. The cumulative distribution function is estimated by plugging them in and denoted by $\hat{G}_{i,t}(\cdot)$. Importantly, the probability of an annual maximum $x_{i,t}$ exceeding some threshold value $q_{i}$ is computed by subtracting the estimated cumulative probability from one

$\Pr\left( x_{i,t}\geq q_{i} \right)=1-\hat{G}_{i,t}(q_{i})=\left\{ \begin{matrix} 1-\exp\left\{ -\left[ 1+\hat{\xi}_{i}\left( \frac{q_{i}-\hat{\mu}_{i,t}}{\hat{\sigma}_{i}} \right) \right]^{-\frac{1}{\hat{\xi}_{i}}} \right\} \mathrm{if} \hat{\xi}_{i}\neq0 \\ 1-\exp\left\{ -\exp\left[ -\left( \frac{q_{i}-\hat{\mu}_{i,t}}{\hat{\sigma}_{i}} \right) \right] \right\} \mathrm{if} \hat{\xi}_{i}=0 \end{matrix} \right.$ (4)

given $1+\hat{\xi}_{i}(q_{i}-\hat{\mu}_{i,t})/\hat{\sigma}_{i}\neq0$. In the following, we denote the $100\alpha\mathrm{th}$ percentile of the annual maxima over the period 1961-1990 of the geographical grid $i$ by $q_{\alpha,i,1961-1990}$. We also use the counterfactual probability in which some component of the radiative forcing is zero. This is computed by using counterfactual location parameter estimate $\hat{\mu}_{i,t}$ in (4), that is, $\hat{\mu}_{i,t}=\hat{\mu}_{0,i}+\hat{\mu}_{1,i}({NAT}_{t})$ when the counterfactual probabilities of no anthropogenic radiative forcings are considered and $\hat{\mu}_{i,t}=\hat{\mu}_{0,i}+\hat{\mu}_{1,i}({GHG}_{t}+{AER}_{t})$ when the counterfactual probabilities of no natural forcing are considered.

The return level that the annual maximum $x_{i,t}$ occurs with probability $p$ is computed by inverting the cumulative probability distribution function of the estimated GEV model as follows

$q_{i,t}=\left\{ \begin{matrix} \hat{\mu}_{i,t}-\frac{\hat{\sigma}_{i}}{\hat{\xi}_{i}}\left[ 1-\left\{ -\log\left( 1-p \right) \right\}^{-\hat{\xi}_{i}} \right], \mathrm{if} \hat{\xi}_{i}\neq0 \\ \hat{\mu}_{i,t}-\hat{\sigma}_{i}\log\left\{ -\log\left( 1-p \right) \right\}, \mathrm{if} \hat{\xi}_{i}=0 \end{matrix} \right.$ (5)

We use $p=1/10$ so that we consider the return level that occurs once in 10 years in each geographical grid $i$. Here, we also use the counterfactual return level by plugging the counterfactual location parameter estimates defined in (5).

In Figure 1, we computed the following change in probabilities and the contributions of the anthropogenic and natural forcings to the probabilities of exceeding the 90^th^ percentile of the reference period (1961-1990) for each grid:

1. $\Pr\left( x_{i,2018}\geq q_{.9, i,1961-1990} \right)-0.1$, for the probabilities of exceedance in 2018. We make a -0.1 correction so that if the series is stationary the value is 0;
2. $\Pr\left( x_{i,2018}\geq q_{.9, i,1961-1990} \right)-\Pr(x_{i,2018}\geq q_{.9, i,1961-1990}|{GHG}_{2018}={AER}_{2018}=0)$, for the contribution of the anthropogenic radiative forcing;
3. $\Pr\left( x_{i,2018}\geq q_{.9, i,1961-1990} \right)-\Pr(x_{i,2018}\geq q_{.9,i, 1961-1990}|{NAT}_{2018}=0)$, for the contribution of the natural radiative forcing.

This approach allows preserving the nonlinearities between the different contributions to the estimated probabilities. In Figure 2, the following changes in return levels of extreme temperature and extreme rainfall are computed:

1. $q_{i} such that\Pr\left( x_{i,2018}\geq q_{i} \right)=0.1$, for the return level in 2018;
2. $\left( q_{i}-q_{ANT,i,2018} \right) such that \Pr\left( x_{i,2018}\geq q_{ANT,i,2018}|{GHG}_{2018}={AER}_{2018}=0 \right)=0.1$, for the contribution of the anthropogenic radiative forcing;
3. $\left( q_{i}-q_{NAT,i,2018} \right) such that \Pr\left( x_{i,2018}\geq q_{NAT,i,2018}|{NAT}_{2018}=0 \right)=0.1$, for the contribution of the natural radiative forcing.

**Supplementary figures**

The supplementary file **Animatedfigures.pptx** contains two animated gif files showing the dynamic evolution of the effect of anthropogenic and natural forcings on the probabilities of exceeding the annual maximum of the extremes for the reference period 1960-1990 across the whole period 1901-2018, that is, for $t=1901, 1902, \ldots, 2018$

$$\Pr\left( x_{i,t}\geq q_{.9, i,1961-1990} \right)-\Pr(x_{i,t}\geq q_{.9, i,1961-1990}|{GHG}_{t}={AER}_{t}=0)$$

for the contribution of the anthropogenic radiative forcing and

$$\Pr\left( x_{i,t}\geq q_{.9, i,1961-1990} \right)-\Pr(x_{i,t}\geq q_{.9,i, 1961-1990}|{NAT}_{t}=0)$$

for the contribution of the natural radiative forcing. This is done for both extreme temperatures and precipitation.
