# Supplementary material for: Anthropogenic influence on extremes and risk hotspots
Source: Sci Rep. 2023 Jan 2;13:35. doi: 10.1038/s41598-022-27220-9 (PMC9807642; doi:10.1038/s41598-022-27220-9)
Supplement: Supplementary file 2 — Supplementary Information 2. [file 41598_2022_27220_MOESM2_ESM.pptx]

## Slide 1
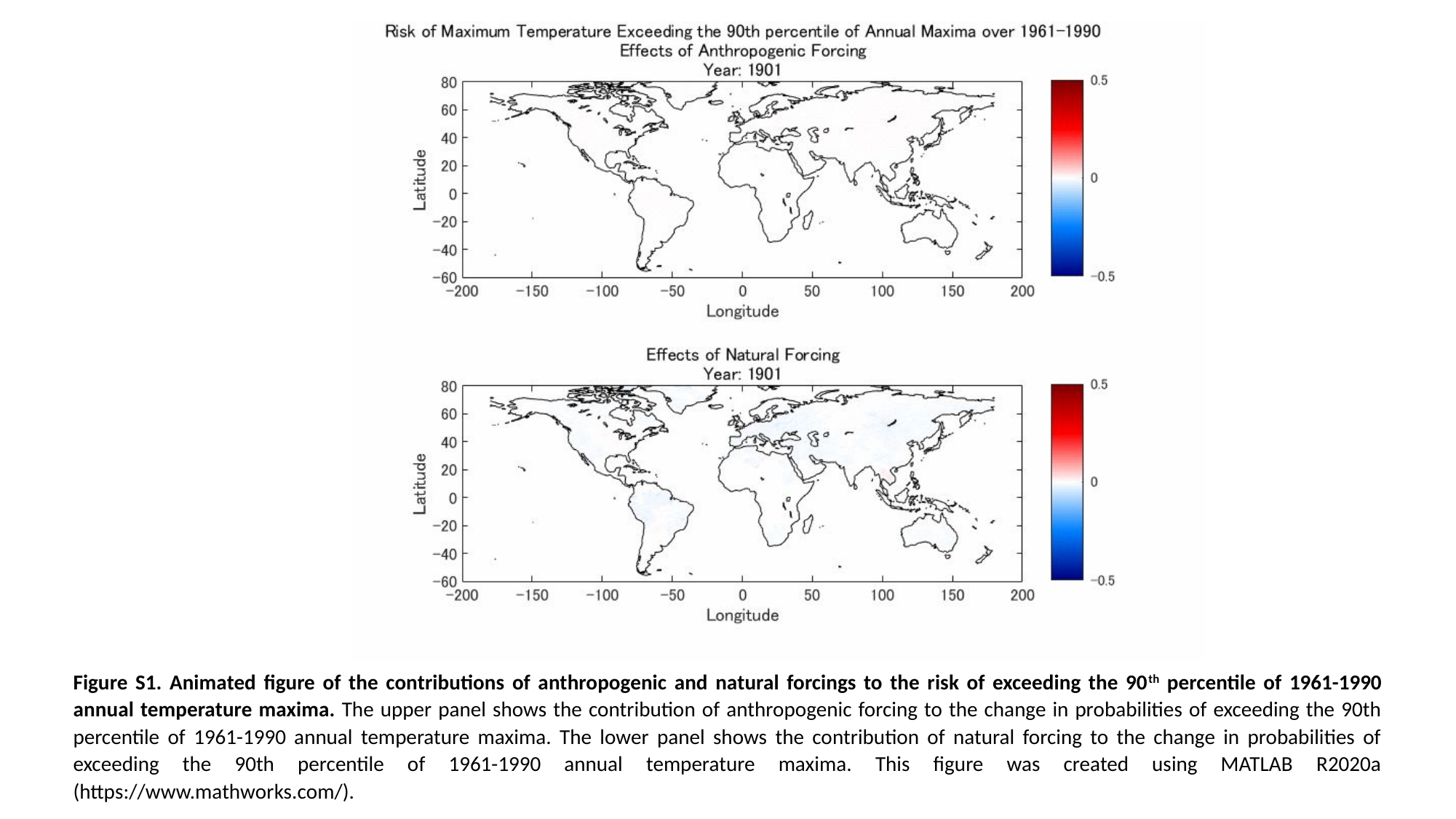

Figure S1. Animated figure of the contributions of anthropogenic and natural forcings to the risk of exceeding the 90th percentile of 1961-1990 annual temperature maxima. The upper panel shows the contribution of anthropogenic forcing to the change in probabilities of exceeding the 90th percentile of 1961-1990 annual temperature maxima. The lower panel shows the contribution of natural forcing to the change in probabilities of exceeding the 90th percentile of 1961-1990 annual temperature maxima. This figure was created using MATLAB R2020a (https://www.mathworks.com/).

## Slide 2
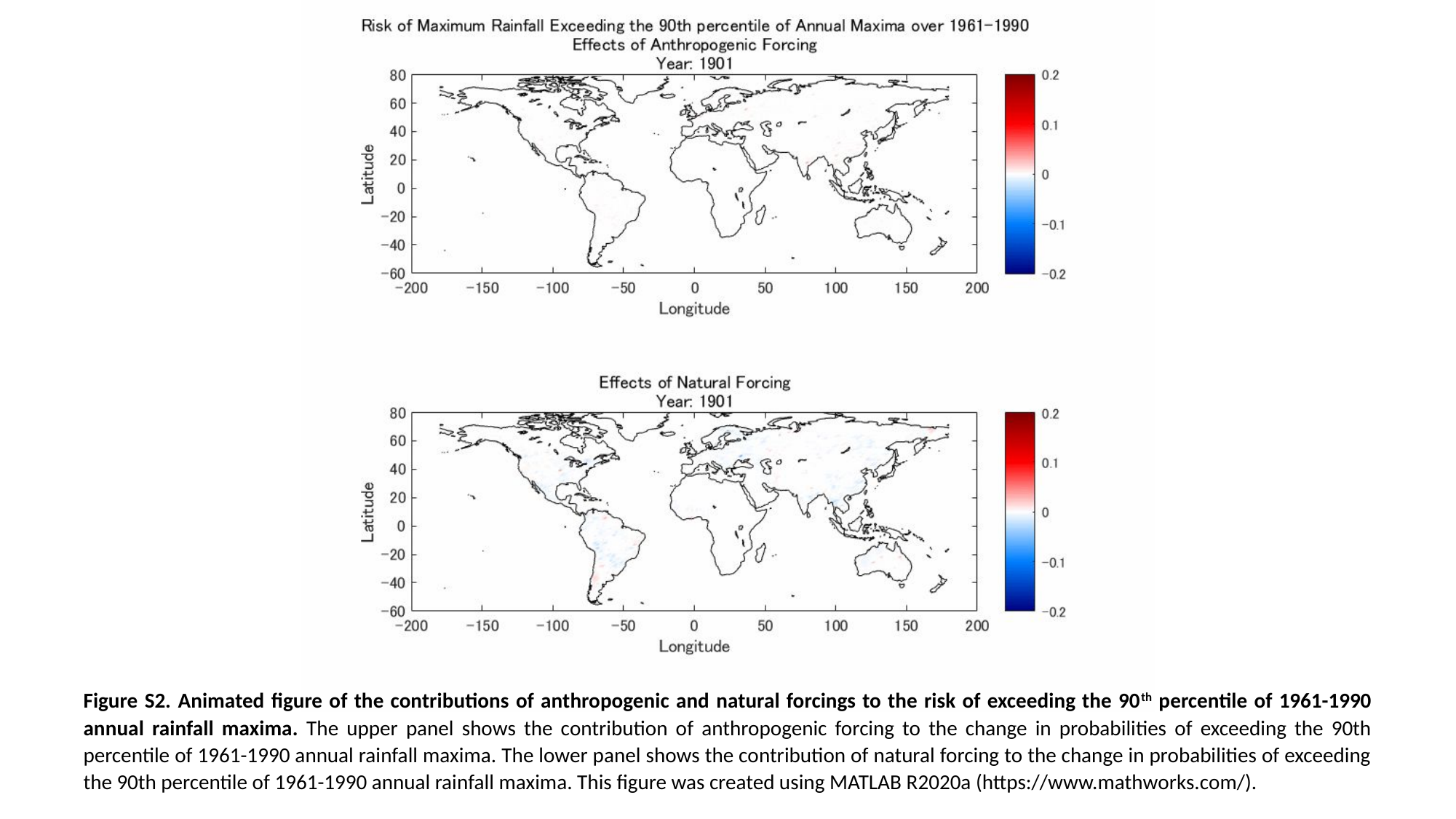

Figure S2. Animated figure of the contributions of anthropogenic and natural forcings to the risk of exceeding the 90th percentile of 1961-1990 annual rainfall maxima. The upper panel shows the contribution of anthropogenic forcing to the change in probabilities of exceeding the 90th percentile of 1961-1990 annual rainfall maxima. The lower panel shows the contribution of natural forcing to the change in probabilities of exceeding the 90th percentile of 1961-1990 annual rainfall maxima. This figure was created using MATLAB R2020a (https://www.mathworks.com/).
